# Plethora from Suppressed Evacuations

**Published:** 1831-04-01

**Authors:** 


### ST. BARTHOLOMEW'S HOSPITAL.

### PLETHORA FROM SUPPRESSED EVACUATIONS.

MR. LAWRENCE, in the report of a clinical lecture in No. 374, of the Lancet, has made some observations on the above subject. He exhibited a specimen of highly inflamed blood taken from a man, "in whom no external evidence of an inflammatory nature existed at the time of its abstraction." The venesection appears to have been performed by the patient's own prescription. This man had been some time in the hospital with a fractured leg, under Mr. Lloyd, before Mr. Lawrence saw him. He had undergone copious general and local depletion, and lived on very low diet. Suppuration had taken place in the fractured limb, and a very copious discharge was the result. Soon after the openings had healed and the discharge had ceased, the patient (ætat. 50) felt himself heated and full, especially about his head. "The man considered himself in a state of plethora and was bled." The blood shewed the true inflammatory characters. The following remarks of Mr. Lawrence are not undeserving of attention.

"It had frequently happened to him, he observed, in this hospital and elsewhere, to see patients who had laboured under profuse local discharges become plethoric, and prone to determinations of blood and internal inflammations when these discharges were suddenly suppressed. This he had seen over and over again under such circumstances in old running ulcers of the leg, more especially if the patients were placed on animal diet and allowed beer, and these determinations would proceed to every degree, up to fatal apoplexy. There was a patient now in 'Henry,' who was in the hospital some years before for inflammation and ulceration of the leg; it was rapidly healed, and in a few days he had a sudden and violent attack of apoplexy; the usual treatment was adopted; he was profusely bled, cupped, leeched, purged, and blistered, but with so little effect, that he (Mr. Lawrence) abandoned all hopes of his recovery; as a last experiment, however, he determined on the employment of mercury in large doses, which was pushed to salivation, and he eventually recovered; but for a long time he was paralytic on one side, and even at present his mouth was somewhat distorted. He had now been seven weeks in the house for a recurrence of the ulceration, and during this time, according as the ulcers were healing, it had been found necessary to bleed him repeatedly, in order to counteract the return of a similar affection to that from which he formerly suffered. The hospital diet table, he was sorry to say, was any-thing but judicious; the 'ordinary diet,' for example, consisted of animal food, two pints of good strong beer—not table beer, but much better —with several other good things. As it is on this diet that a patient is placed on admission, and as it may happen that a patient may not be seen immediately after entrance, and mean while may labour under acute inflammation, this diet would, of all other things, be the best calculated to aggravate his disease. He had, therefore, made it a general rule, that his patients should be placed, at first, on milk diet, for if they were in a condition for beer and meat, the hospital was not the fittest place for their reception."

